# Impact of an intrapartum care quality improvement intervention in Brazilian private hospitals on care safety measures and adverse outcomes

**DOI:** 10.1186/s12978-022-01541-4

**Published:** 2023-02-02

**Authors:** Maíra Libertad Soligo Takemoto, Marcos Nakamura-Pereira, Fernando Maia Peixoto-Filho, Maria do Carmo Leal

**Affiliations:** 1 Botucatu Medical School, Universidade Estadual Paulista Júlio de Mesquita Filho, UNESP - Campus de Botucatu, Av. Prof. Mário Rubens Guimarães Montenegro, s/n , Botucatu, SP 18618-687 Brazil; 2 National Institute of Health for Women, Children and Adolescents Fernandes Figueira (IFF/Fiocruz), Av. Rui Barbosa, 716 - Flamengo, Rio de Janeiro, RJ Brazil; 3 Oswaldo Cruz Foundation, National School of Public Health, Leopoldo Bulhões Street, 951, 8º Floor, Bonsucesso, Rio de Janeiro, RJ 21041-210 Brazil

**Keywords:** Cesarean section, Quality improvement, Obstetrics, Maternal Health, Health Services Research, Evaluation Study

## Abstract

**Background:**

In 2015, a quality improvement (QI) intervention to reduce cesarean sections (CS)—the Adequate Childbirth Project (PPA)—was implemented in the private sector in Brazil. This analysis aims to compare safety care measures and adverse outcomes between women exposed to the PPA intervention to those receiving standard care.

**Methods:**

The analysis included a convenience sample of 12 private hospitals that participated in the PPA (2017–2018). Data collection was performed through chart review and interviews. Differences in 15 outcomes were examined using Pearson’s chi-square test and multiple logistic regressions.

**Results:**

The final weighted sample was comprised of 4789 births, 2570 in the PPA group (53.5%) and 2227 in the standard care group (46.5%). CS rate was significantly lower in the PPA group (67.3% vs 88.8%). After adjusting for potential confounders, PPA model was associated with decreased overall CS rate (OR = 0.30, 95% CI 0.24 to 0.36), as well as prelabor (OR = 0.41, 0.34 to 0.48) and repeated CS (OR = 0.45, 0.29 to 0.70). In terms of other safety care measures, women in the PPA model had an increased chance of absence of antibiotic prophylaxis in Group B Streptococcus (GBS) + women (OR = 4.63, 1.33 to 16.14) and for CSs (OR = 1.75, 1.38 to 2.22), while those with severe hypertension were less likely to not receiving magnesium sulphate (OR = 0.27, 0.09 to 0.77). Regarding obstetric and neonatal outcomes, PPA model was associated with a decreased chance of having an obstetric anal sphincter injury (OASI) following an episiotomy (OR = 0.34, 0.13 to 0.89), requiring antibiotics other than routine prophylaxis (OR = 0.84, 0.70 to 0.99), having a late preterm (OR = 0.36, 0.27 to 0.48) or early term baby (OR = 0.81, 0.70 to 0.94). There were no statistically significant differences for other outcomes.

**Conclusions:**

The PPA intervention was able to reduce CS rates, late preterm and early term deliveries without increasing the chance of adverse outcomes. The bidirectional effect on safety care measures reinforces that QI initiatives includes closer observation of routine care when implementing interventions to reduce C-section rates.

## Plain Language Summary

Cesarean section rates in Brazil are among the highest in the world, particularly in private hospitals. In 2015, a quality improvement project was implemented in private hospitals aiming to reduce the cesarean section (CS) rates (the Adequate Childbirth Project—PPA). In the 2017–2018 period, the Healthy Birth Study (HBS) was proposed to assess the effect of the PPA project in CS rates, as well as use of obstetric interventions, adoption of good practices during labor and birth care and outcomes for both women and their babies. This article presents the comparison of 4873 births analyzed in the HBS, 2589 who were exposed to the PPA project and 2284 who received standard care. The analysis aim was to compare CS rates at the same time to assess if women who were part of the PPA intervention were less likely to have a negative event for themselves or their babies. A group of 15 measures of safety of obstetric care and negative outcomes for women and their babies was compared. Women who were exposed to the PPA intervention had a lower chance of CS, late preterm and early term deliveries. At the same time, the PPA group did not have worse outcomes for women or babies. In terms of safety care, the PPA intervention was associated with both positive and negative effects. For instance, women exposed to the PPA group had a higher chance of not receiving antibiotics to prevent infections when they needed, when compared to standard care.

## Background

Cesarean section (CS) rates are increasing worldwide in response to several factors including clinical, cultural, and financial aspects [1]. Brazilian CS rates are internationally recognized as alarming and have steadily increased since the 1980s [2–5]. The private sector in Brazil accounts for the highest CS rates (reaching about 90% of all births in some scenarios) [3, 6] and the association between source of payment and increased use of CS is also observed in other countries [7]. Thus, public policies and strategies aiming to reduce CS rates in the country need to notably address the issue within the private sector.

In 2015, in response to social and legal pressure, the Brazilian Ministry of Health regulatory agency responsible for regulating and inspecting private health insurance companies proposed a quality improvement (QI) intervention named “*Parto Adequado*” (PPA, Adequate Childbirth Project in English) [8–10]. PPA aimed to identify innovative and feasible models of care for labor and childbirth focused on encourage vaginal births and reduce rates of cesarean sections without medical indications in the private healthcare system in Brazil [8–10]. The PPA model was implemented in three phases: Phase 1 was implemented in 2015–2016 with the goal to test the QI intervention and enrolled 35 public and private maternity hospitals and 19 private insurance companies; Phase 2 started in 2017 and is still ongoing and aimed to expand the project to more maternity hospitals and insurance companies; Phase 3 was launched in 2019 expecting to disseminate strategies to improve quality of labor and childbirth care in Brazil on a larger scale, potentially including all Brazilian maternity hospitals and insurance companies in Brazil [8, 9].

The PPA QI intervention has four main theoretical driving components: (i) coalition building between different stakeholders within the health system with a common purpose of improve quality and safety in labor and childbirth care; (ii) empowering pregnant women and families to actively participate in the care from pregnancy to postpartum; (iii) implementation of innovative models of care favoring physiologic birth and cesarean section decision based on clinical indications; (iv) implement and improve information systems to gather information that allow for continuous learning. For each of the four driving components, the PPA proposed several strategies and actions to be implemented by the hospitals that joined the project. The PPA initial goal was to decrease cesarean sections while not increasing perinatal risks and overall adverse outcomes [8–10].

In 2017, a hospital-based, cross-sectional, evaluative research was proposed to evaluate the degree of implementation of PPA as well as its impact on obstetric and neonatal outcomes in 12 maternity hospitals that joined PPA in its Phase 1—the Healthy Birth Study (HBS) [8]. The present analysis is part of the HBS evaluative research and aimed to examine the effect of PPA on safety care measures [11, 12] and obstetric and neonatal outcomes. We hypothesize that being exposed to the PPA model of care would reduce the chance of a woman experience a negative outcome or an unsafe care event.

## Methods

### Healthy Birth Study (HBS)

The HBS was a hospital-based, cross-sectional, evaluative research conducted from March to August 2017, e.g., six to eight months after the PPA intervention first phase implementation [8]. A convenience sample of 12 out of the 23 private hospitals that participated in the PPA first phase was selected based on the following criteria: Brazilian geographic macro-region (South/Southeast/Midwest and North/Northeast); type of hospital (hospitals owned or not owned by health insurance companies); and hospital performance in implementing PPA intervention according to information provided by the project coordination team. Within each hospital, a 400 women sample was calculated to detect a 10% reduction in the CS rate, considering an expected prevalence of 50%, 80% of power and a level of significance of 5%. More information on the sample size calculation and sampling strategy can be found in Torres et al. 2018 [8].

Women were deemed eligible if they had given birth to a live born in any gestational age or birth weight or had a stillbirth with ≥ 22 weeks or ≥ 500 g. Exclusion criteria were: hearing impairment; foreign women who did not speak Portuguese; multiple pregnancies with 3 or more fetuses; and women admitted to legal termination of pregnancy. No age restriction criteria were applied. Eligible women were consecutively invited to participate in the study irrespective of delivery mode until the planned sample size was reached.

The HBS primary outcome was the overall CS rate. Secondary outcomes included: (i) CS rate by Robson Classification Groups [6, 13]; (ii) woman’s satisfaction with care; (iii) severe maternal morbidity and maternal near-miss according to the World Health Organization criteria [14]; (iv) proportion of preterm (gestational age < 37 weeks) and early term (37 and 38 weeks) births; and (v) neonatal intensive care unit admission, neonatal near miss and perinatal mortality.

Data collection was performed using multiple sources depending on the type of data: interviews with hospital director or head of Obstetrics or Nursing to collect data on hospital infrastructure and work process; face to face interviews with postpartum women about demographics, obstetric and medical history, prenatal care, labor and birth events, self-reported evaluation of both woman and newborn care during hospital stay; and medical charts review to collect data on labor, birth and newborn care including tests, procedures, interventions and diagnosis. Detailed information on data collection and variables, including all the data collection instruments and the interview guide, was published elsewhere [8].

The present analysis aimed to assess the impact of the PPA model in a set of safety care measures and obstetric and neonatal outcomes by comparing these outcomes between women exposed to the PPA model or to standard care, using the HBS database.

### Exposure variables

For the purposes of the present analysis, the sample was classified according to the exposure to the model of care proposed by the PPA: PPA model (those exposed to the PPA intervention) and standard care (non-exposed to the PPA intervention). Criteria for taking part in the PPA model of care were defined by each hospital (for example, all Robson Groups 1–4 women or all women cared for by the hospital staff and not a private independently contracted care provider) and the HBS study variable “model of care” was collected considering the hospital criteria. Women in the PPA model were exposed to interventions proposed in the QI project, such as antenatal classes, visit to the hospital before labor, incentive to prepare a birth plan, intrapartum care in a collaborative model between obstetricians and midwives, and use of best practices during labor and birth. The standard care group received the usual practices commonly offered within private maternity hospitals in Brazil, typically characterized by intrapartum care provided by the same obstetrician who provided antenatal care, reduced participation of midwives, increased proportion of antepartum cesarean sections, and intensive use of interventions during labor and birth.

In the PPA first phase (object of this analysis), each hospital established the criteria to select the PPA target population. The PPA target population was defined as: all nulliparous women in two hospitals; Robson Classification Groups 1 to 4 in two hospitals; and all women admitted for labor and delivery by the hospital staff (and nor their antenatal obstetrician) in 8 hospitals (one hospital also required that women be from the Robson Classification Group 1 to 4, and another one required that they did not have a previous uterine scar). This variety of criteria to select women to the QI intervention likely produced between-groups differences in terms of characteristics that would also impact the safety care measures and outcomes assessed in the present analysis.

To account for these differences, the following additional exposure variables were selected: preterm pregnancy at admission or birth, high-risk pregnancy, previous cesarean section, previous vaginal birth, type of pregnancy (single or twin), fetal presentation at admission (cephalic or non-cephalic). High-risk pregnancies were defined as those in women who presented one or more of the following conditions: gestational hypertension/pre-eclampsia, chronic hypertension, eclampsia, pre-gestational diabetes, gestational diabetes, severe chronic diseases, infection at the time of admission for childbirth (including urinary tract infection and other severe infections, such as chorioamnionitis and pneumonia), placental abruption, placenta previa, intrauterine growth restriction, and known fetal anomalies.

### Outcomes

Besides HBS main outcome (cesarean section rate), two sets of outcomes were selected to explore the impact of the PPA model: safety care measures and obstetric or neonatal outcomes. These sets of outcomes were largely based in outcome measures proposed in obstetric safety care studies, as well as obstetric care quality improvement initiatives [12, 15, 16]. The following measures were analyzed: Prelabor c-section; Repeated c-section (among women with at least one previous cesarean section); Prelabor c-section in women with prelabor rupture of membranes (PROM); Amniotomy during labor < 6 cm of cervical dilation; Group B Streptococcus positive women without prophylaxis during labor; C-section without antibiotic prophylaxis; Absence of postpartum (PP) prophylactic oxytocin; Severe hypertension without magnesium sulphate; Rhesus negative mother with a Rhesus positive baby without anti-D prophylaxis; and preterm birth < 34 weeks of gestational age without corticosteroids.

Selected obstetric and neonatal outcomes were: Severe postpartum hemorrhage; Return to the Operating Room or Labor & Delivery unit after birth due to complications; Intraoperative injury (among women who had undergone cesarean sections only); Uterine rupture; obstetric anal sphincter injuries (OASIS); Episiotomy with OASIS; Antibiotics use (excluding prophylaxis); Severe maternal morbidity; Maternal intensive care unit (ICU) admission; Late preterm birth (34–36 weeks); Early term birth (37–38 weeks); Neonatal birth trauma; 5 min Apgar < 7; Neonatal ICU admission (among babies born > 36 weeks); and Neonatal death (among babies born > 36 weeks).

### Data analysis

To compare between-groups differences in outcomes, Pearson’s chi-square test, adjusted for the survey design using STATA “svy” commands, was employed for bivariate analysis (0.05 significance level). All outcomes were additionally explored using logistic regression using STATA svy: logistic command with exposures variables included in each model depending on the specific outcome (a priori selected). Logistic regression models were adjusted for model of care and all other aforementioned exposure variables with the following exceptions: previous cesarean section was not included in the repeated cesarean section model and preterm pregnancy at admission was not included in late preterm birth, early term birth, neonatal ICU admission > 36 weeks, and neonatal death > 36 weeks. Odds ratio and their 95% Confidence Intervals (CIs) were calculated and reported and p-value < 0.05 was considered statistically significant. All analyses were performed considering the design effect weights of the HBS using STATA/MP 16.1 (STATA LLC, College Station, TX, US) survey data analysis features.

## Results

The final weighted sample was comprised of 4,789 births, 2,570 in the PPA group (53.5%) and 2, 227 in the standard care group (46.50%). Statistically significant differences in baseline characteristics were observed for all variables except ethnicity/skin color and at least one previous vaginal birth (Table 1). Women in the PPA group were slightly younger, more frequently nulliparous, without a previous c-section, with a cephalic presentation at admission. They were also less likely to have either a twin or preterm pregnancy at admission. The frequency of high-risk pregnancy was slightly higher in the standard care group.

**Table 1:** Sample characteristics according to model of care (PPA model vs standard care)

	PPA (n = 2570)		Standard care (n = 2227)		p-value*
	n	%	n	%	
Age					
13–19 years	67	2.6	16	0.7	**< 0.001**
20–34 years	1753	68.2	1315	59.0	
35–39 years	596	23.2	746	33.5	
40+ years	154	6.0	151	6.8	
Ethnicity/skin color					
White	1733	67.4	1506	67.6	
Black	136	5.3	93	4.2	0.241
Brown/yellow/indigenous	703	27.3	627	28.2	
Nulliparous	2084	81.1	762	34.2	**< 0.001**
At least one previous vaginal birth	327	12.7	281	12.6	0.856
Previous c-section	182	7.1	1246	55.9	**< 0.001**
High-risk pregnancy	514	20.0	532	23.9	**0.005**
Twin pregnancy	18	0.7	65	2.9	**< 0.001**
Cephalic presentation at admission	2462	95.7	1981	88.9	**< 0.001**
Preterm at admission	199	7.7	331	14.8	**< 0.001**

*Design-based Pearson's chi-squared test

Table 2 presents the bivariate analysis of selected safety care measures and outcomes. C-section rate was significantly lower in the PPA group (67.3% vs 88.8%), as were prelabor c-section (53.1% vs 77.1%), repeated c-section (84.8% vs 92.7%) and prelabor c-section due to PROM at admission (36.0% vs 50.8%) rates. In terms of safety care measures, statistically significant differences in favor of the standard care group were observed for absence of antibiotic prophylaxis among Group B Streptococcus (GBS) positive women in labor (8.0% vs 1.6%) and during cesarean sections (15.8% vs 11.8%). Absence of magnesium sulphate in severe hypertension cases was more frequent in the standard care group (30.8% vs 56.7%).

**Table 2:** Bivariate analysis of safety care measure and obstetric or neonatal outcomes according to model of care (PPA model vs standard care)

	PPA		Standard care		p-value*
	n		n		
C-section	1728	67.3%	1970	88.8%	**< 0.001**
Safety care measures					
Prelabor c-section	1364	53.1%	1717	77.1%	**< 0.001**
Repeated c-section	153	84.8%	1154	92.7%	**< 0.001**
Prelabor c-section in PROM	137	36.0%	125	50.8%	0.068
Amniotomy during labor < 6 cm	37	1.4%	6	0.3%	0.119
GBS + without prophylaxis during labor	32	8.0%	5	1.6%	**0.016**
C-section without antibiotic prophylaxis	273	15.8%	232	11.8%	**< 0.001**
Absence of PP prophylactic oxytocin	310	12.1%	248	11.1%	0.149
Severe hypertension without MgSO4	14	30.8%	18	56.7%	**0.014**
Rh− mother / Rh + baby without anti-D	53	28.4%	48	29.2%	0.227
Preterm < 34w birth without corticosteroids	18	35.8%	28	48.6%	0.308
Obstetric and perinatal outcomes					
Severe postpartum hemorrhage	19	0.8%	18	0.8%	0.681
Return to OR / L&D unit	16	0.6%	10	0.5%	0.405
Intraoperative injury (c-section only)	25	1.4%	41	2.1%	0.878
Uterine rupture	1	0.0%	3	0.1%	0.166
OASIS	145	17.2%	60	24.0%	0.142
Episiotomy with OASIS	9	2.5%	5	7.5%	**0.028**
Antibiotics use (excluding prophylaxis)	562	21.9%	601	27.1%	**0.039**
Severe maternal morbidity	102	4.0%	105	4.7%	0.719
Maternal ICU	34	1.3%	28	1.2%	0.206
Late preterm	148	5.7%	273	12.2%	**< 0.001**
Early term	854	33.2%	916	41.1%	**0.005**
Neonatal birth injury	40	1.6%	39	17.9%	0.705
Apgar 5 min < 7	18	0.7%	13	0.6%	0.877
Neonatal ICU admission > 36w	137	5.7%	126	6.3%	0.393
Neonatal death > 36w	1	0.0%	2	0.1%	0.354

PROM: premature rupture of membranes; GBS: Groups B Streptococcus; PP: postpartum; MgSO4: magnesium sulphate; OR/L&D: operating room or labor and delivery unit; OASIS: obstetric anal sphincter injuries; ICU: intensive care unit

*Design-based Pearson's chi-squared test

In the bivariate analysis, regarding obstetric and perinatal outcomes (Table 2), women in the PPA group had lower frequency of OASI after an episiotomy (2.5% vs 7.5%), antibiotics use during their hospital stay excluding prophylactic antibiotic (21.9% vs 27.1%), as well as late preterm (5.7% vs 12.2%) and early term births (33.2% vs 41.1%).

**Fig. 1:**
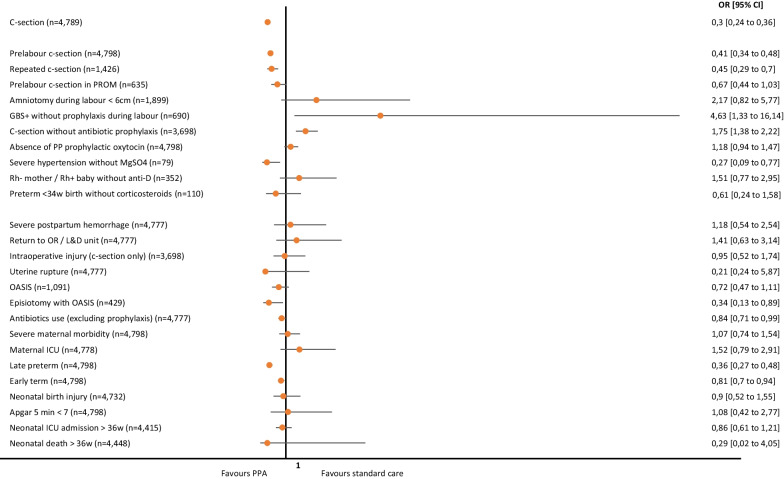
– Adjusted* Odds Ratio and 95% Confidence Intervals for each safety care measure and obstetric or neonatal outcomes according to model of care. OR: odds ratio; CI: confidence interval; PROM: premature rupture of membranes; PP: postpartum; MgSO4: magnesium sulphate; OR/L&D: operating room or labor and delivery unit; OASIS: obstetric anal sphincter injuries; ICU: intensive care unit. *Models were adjusted for the following exposure variables: preterm pregnancy at admission or birth, high-risk pregnancy, previous cesarean section, previous vaginal birth, type of pregnancy (single or twin), fetal presentation at admission (cephalic or non-cephalic), with the following exceptions: previous cesarean section was not included in the repeated cesarean section model and preterm pregnancy at admission was not included in late preterm birth, early term birth, neonatal ICU admission > 36 weeks, and neonatal death > 36 weeks

Figure 1 presents adjusted Odds Ratio (OR) and 95% Confidence Interval (CI) for each outcome following multiple logistic regression. After adjusting for potential confounders, all significant outcomes in the bivariate analysis remained significant except for c-section without labor for women with PROM at admission. PPA model was associated with decreased overall c-section rate (OR = 0.3, 95% CI 0.24 to 0.36), as well as prelabor (OR = 0.41, 0.34 to 0.48) and repeated c-section (OR = 0.45, 0.29 to 0.70). Conversely, an increased chance of absence of antibiotic prophylaxis in GBS + women (OR = 4.63, 1.33 to 16.14) and for c-sections (OR = 1.75, 1.38 to 2.22) was associated with being in the PPA group. Women in the PPA model also had less chance of not receiving magnesium sulphate if they had severe hypertension (OR = 0.27, 0.09 to 0.77), having and OASIS following an episiotomy (OR = 0.34, 0.13 to 0.89), requiring antibiotics other than routine prophylaxis (OR = 0.84, 0.71 to 0.99), having a late preterm (OR = 0.36, 0.27 to 0.48) or early term baby (OR = 0.81, 0.70 to 0.94).

## Discussion

Our findings indicate that the PPA QI project was successfully able to reduce overall cesarean section rates, also reducing cesarean sections performed before labor and in women with previous uterine scars, as well as late preterm and early term births. It was not possible to see a clear direction of the effect on safer care during labor and birth, once a few of the unsafe care events were actually more common in the PPA group than in the standard care group. Also, the significant reduction in cesarean section rates was not followed by increments in any of the assessed adverse obstetric and perinatal outcomes, reaching the initial goal of the PPA project [9].

Preliminary results for the PPA intervention were initially published in 2019, reporting findings from an analysis comparing HBS findings to data from all private hospitals enrolled in a nationwide survey that collected data in 2011–2012 (Birth in Brazil Survey), thus before PPA project implementation. A reduction in cesarean sections before labor and a resulting increase in intrapartum cesarean section was reported for the Brazilian private sector from 2011–2012 to 2017 (from 5.5% to 13.6%), with an accompanying increase of 85% in the vaginal delivery rate (from 12.3% to 22.8%) [10]. In 2020, another study reported an increase in vaginal delivery from 21.5% in 2014 to 34.8% in 2016 in 13 hospitals with complete data at that time [9]. Also, despite the fact that these findings were not before and after comparisons of the same 12 hospitals enrolled in the HBS, they provided a preliminary overview of PPA model impact in CS rates.

While reducing CS rate is a valid and widely used goal for obstetric QI initiatives, the risk of increasing adverse outcomes may be a concern for clinicians and the society, mostly in settings where cesarean sections are culturally considered safe as in Brazil. Trying to respond to these concerns, we assessed 15 obstetric and perinatal outcomes and were not able to identify negative effects of the PPA QI in any of them. In fact, women in the standard care group were more likely to receive antibiotics other than routine prophylaxis (a proxy of an infection event), have an OASIS following an episiotomy, and have their babies more frequently delivered late preterm or early term. Adverse perinatal outcomes were comparable among groups and the PPA model was not associated with an increase in 5-min Apgar score < 7, admission to neonatal ICU or death.

Available studies assessing quality improvement projects in Obstetrics present significant differences in terms of sample characteristics, interventions and outcomes, thus comparisons of findings are challenging [17–21]. A US study published in 2017 reported the results of a QI project implemented in a community hospital in Colorado, aiming to reduce the primary CS rate [21]. The focus of this QI project were 3 strategies to promote physiologic birth: reducing induction of labor in pregnancies before 41 weeks gestation, admission in labor only with ≥ 4 cm dilation, encourage the use of intermittent auscultation rather than continuous electronic fetal monitoring. The primary CS rate was reduced from 28.9% to 12.2% after a 12-month period (OR = 0.34, 0.25 to 0.48). The observed OR was quite like the one estimated in our analysis, even with a baseline CS rate markedly lower than the one from the standard care in HBS regardless of parity (28.9% vs 88.9%).

A Statewide QI project conducted in Ohio, US, to reduce early elective deliveries at less than 39 weeks of gestation enrolled 72 hospitals from February 2013 to March 2014. The QI intervention focused on educational strategies and was based on a learning collaborative model and individual quality improvement coaching. Kaplan et al. reported a statistically significant decline in nonmedically indicated inductions of labor before 39 weeks of gestation after implementation (absolute difference of 2–3% in the before after analysis performed in 3 different time points) [19]. A similar Statewide QI initiative was also implemented in New York, US, in 2012–2014 [20]. Non-medically indicated scheduled CS at 36–38 weeks gestation reduced from 18.9% at baseline to 0.4% at the end of the project, while inductions of labor declined from 4.2% to 0.4%. The results of a single-center obstetric QI project developed between 2014 and 2016 were reported by Ogunyemi et al. [17] using a multi-faceted intervention addressed 3 main dimensions: assessing institutional culture, improving education and awareness, and optimizing obstetric system process. The primary CS rate in singleton vertex pregnancies significantly reduced from 23.4% to 14.1% and the rate among nulliparous singleton term vertex pregnancies decreased from 34.5% to 19.2%. The authors also observed a decline in neonatal ICU admission without adverse impact in other maternal and perinatal outcomes, similar to our findings [17].

A secondary analysis of the *Birth in Brazil* study identified a high prevalence of preventable harm in the context of obstetric care in the Southeast region of the country, with only 2% of women classified as harm-free during childbirth [22]. Specifically in the private sector, CS births before labor presented a fourfold increase in the odds of having one or two or more harms. Thus, the reduction in overall CS and prelabor CS rates would be expected to improve safety measures. Further, an obstetric QI project including interventions targeting stakeholders’ culture, changes in the management level of institutions, and educational interventions would also be expected to improve the adoption of obstetric safety practices and reduce unsafe care events. However, it was not possible to identify a clear effect on safety care measures in the present analysis. These findings highlight the need to include strategies to improve the adoption of evidence-based practices associated with increased obstetric safety alongside QI projects aiming to reduce cesarean section rates [12, 16].

It is not possible to examine the potential causes of the increased likelihood of some unsafe care events in the PPA group by exploring the HBS database. However, it is possible to hypothesize some likely explanations. One of the strategies developed within PPA to reorganize obstetric care was to move from intrapartum care provided by the same obstetrician from prenatal through labor to intrapartum care provided by a hospital team [8–10]. In the latter model, providers are hired by the hospital and are maybe more likely to follow institutional guidelines, which may explain the higher chance of receiving magnesium sulphate for severe hypertension. The higher participation of intrapartum CS as opposed to scheduled prelabor CS in the PPA model may partially explain the decline in perioperative antibiotic prophylaxis usage, due to less routine, structured care paths in non-elective surgical procedures. It is worth highlighting that obstetric outcomes that could have been affected by this difference in safety care measures were not worsened by the PPA intervention in our analysis, particularly severe postpartum hemorrhage, antibiotics use (excluding prophylaxis), and severe maternal morbidity.

Our study presents some important limitations. First, the study included a convenience sample with specific eligibility criteria [8]. Thus, HBS findings may not represent all hospitals that joined the PPA project. Differences in baseline characteristics that are known to influence the cesarean section risk were observed between PPA and standard care groups, thus data analysis adjustments were made. However, it is not possible to rule out that the different eligibility criteria to be exposed to the PPA QI intervention in each hospital may have led to differences in baseline characteristics that were not totally possible to account for by using logistic regression adjustments. Additionally, for some of the outcomes the subgroup with available data had a small sample size, which may have impaired our ability to observed differences that larger samples sizes would reveal. Despite these limitations, the HBS large sample size and set of variables allow a comprehensive overview of PPA model results, including its impact in several outcomes simultaneously. These findings raise relevant hypothesis for further investigation regarding the effects of QI interventions to reduce unnecessary cesarean sections.

## Conclusions

The Adequate Childbirth Project Quality Improvement intervention was able to reduce cesarean section rates without increasing the chance of adverse obstetric and perinatal outcomes. The bidirectional effect on obstetric safety care measures reinforces that QI initiatives include closer observation of routine and responsive care when implementing interventions to reduce unnecessary C-section, focusing on strategies to disseminate evidence-based practices associated with increased care safety.

## Data Availability

The datasets generated and/or analysed during the current study are available in the "Nascer Saudável" repository, 10.35078/C1PSMZ.

## Abbreviations

CI: Confidence Interval
CS: Cesarean section
GBS: Group B Streptococcus
HBS: Health Birth Study
ICU: Intensive Care Unit
L&D: Labor and Delivery Unit
OR: Odds ratio
PPA: Adequate Childbirth Project (Projeto Parto Adequado)
PROM: Prelabor rupture of membranes
QI: Quality improvement
